# The Reduced Graphene Oxide (rGO) Induces Apoptosis, Autophagy and Cell Cycle Arrest in Breast Cancer Cells

**DOI:** 10.3390/ijms23169285

**Published:** 2022-08-18

**Authors:** Rafał Krętowski, Marzanna Cechowska-Pasko

**Affiliations:** Department of Pharmaceutical Biochemistry, Medical University of Białystok, Mickiewicza 2A, 15-222 Białystok, Poland

**Keywords:** autophagy, apoptosis, breast cancer cells, nanotoxicity, reduced graphene oxide, rGO

## Abstract

Reduced graphene oxide (rGO) has already been reported as a potential cytostatic agent in various cancers. However, the mechanisms underlying rGO’s cytotoxicity are still insufficiently understood. Thus, the aim of the study was to investigate the molecular and cellular effects of rGO in breast cancer. Given this, two cell lines, MDA-MB-231 and ZR-75-1, were analyzed using MTT test, flow cytometry and Western blot assay. Incubation with rGO resulted in a multitude of effects, including the stimulation of autophagy, cell cycle arrest and, finally, the apoptotic death of cancer cells. Notably, rGO had minimal effect on normal human fibroblasts. Apoptosis in cancer cells was accompanied by decreased mitochondrial membrane potential, the deregulated expression of mitochondrial proteins and the activation of caspase 9 and caspase 3, suggesting that rGO predominantly induced apoptosis via intrinsic pathway. The analysis of LC3 protein expression revealed that rGO also caused autophagy in breast cancer cells. Moreover, rGO treatment resulted in cell cycle arrest, which was accompanied by deregulated p21 expression. Altogether, rGO seems to have multidirectional cytostatic and cytotoxic effects in breast cancer cells, making it a promising agent worthy of further investigation.

## 1. Introduction

Globally, cancer is one of the primary causes of mortality [1]. In 2008, 8 million cancer deaths were recorded, and this figure is estimated to reach 11 million by 2030 [2]. Breast cancer is a highly heterogeneous cancer encompassing a group of genetically and epigenetically distinct diseases and is the biggest oncological problem in developed countries [3,4]. Breast cancer is the most often diagnosed cancer and the leading cause of cancer death among females, accounting for 23% of the total cancer cases [5].

Graphene is isolated from crystalline graphite Graphene is a flat monolayer composed of a single atom thick, two-dimensional sheet of a hexagonally arranged honeycomb lattice [6]. Graphene-based nanomaterials (GNM) include nanodimensional flakes or ribbons of pure graphene, graphene oxide (GO) and reduced graphene oxide (rGO) all consisting of single graphene layers [7,8]. GNM have various applications and are generally used in the biomedical field in diagnostics, bio-imaging and drug delivery systems. GNM present in biomedical and non-biomedical seller have shown potential toxicity to cancer cells [9,10,11,12,13,14,15,16,17]. rGO has more oxygen groups than graphene but fewer oxygen groups than GO. Ergo, rGO is less hydrophilic unto the GO but also has a higher electrical conductivity [18]. The primary cause of rGO cytotoxicity is the interaction of hydrophobic rGO with cell membranes. rGO easily enter the living cells due to their small size, sharp edges and rough surface [6]. 

The interactions of rGO with cells can lead to membrane damage and cytotoxicity [19]. Several studies have confirmed the antitumor effect of GO through targeting drug carriers [20]. Graphene materials resulted in various degrees of cell apoptosis, necrosis or autophagy [6]. The GO and rGO treatments resulted in cell apoptosis of A549 and HUVEC cells and the functionalization of the investigated GNM decreased cytotoxicity and apoptosis [6]. Graphene quantum dots (GQDs) induced autophagy in SGC-7901 cells through the mTOR signaling pathway [19]. Moreover, Qin et al. indicated that p38 MAPK and NF-κB pathways were concernedin GQD-induced apoptosis and autophagy of macrophages [21]. 

In our previous studies investigating breast cancer cell lines treated with rGO, we indicated that rGO can induce cytotoxicity and reduce proliferation time in a dose-dependent manner in MDA-MB-231 and ZR-75-1 cells but not in T-47D, MCF-7 and Hs 578T cells. Moreover, in cells which responded to treatment with rGO, we observed increased oxidative stress, the upregulation of apoptosis, the accumulation of nuclear condensation and the marginalization of chromatin [22].

Apoptosis and autophagy are well known, highly conserved processes, which are involved in tumorigenesis, inflammation and cellular senescence. Autophagy and apoptosis are both cellular pathways, which are essential for organismal homeostasis and serve as a main target of tumor therapeutics [6,23]. The molecular connections between autophagy and apoptosis are multifaceted, complex and still poorly understood. The process of autophagy could control apoptosis and vice versa [24].

Apoptosis and autophagy can be regulated by graphene nanoparticles [19]. A small number of publications are concerned with the influence of rGO on the mechanism of apoptosis or autophagy in breast cancer cell lines. Therefore, in our reaearch, we investigate the mechanisms of apoptosis and autophagy in breast cancer cell lines incubated with rGO.

## 2. Results

### 2.1. rGO Induced Cytotoxicity of MDA-MB-231 and ZR-75-1 Cells in Dose-Dependent Manner

**Figure 1:**
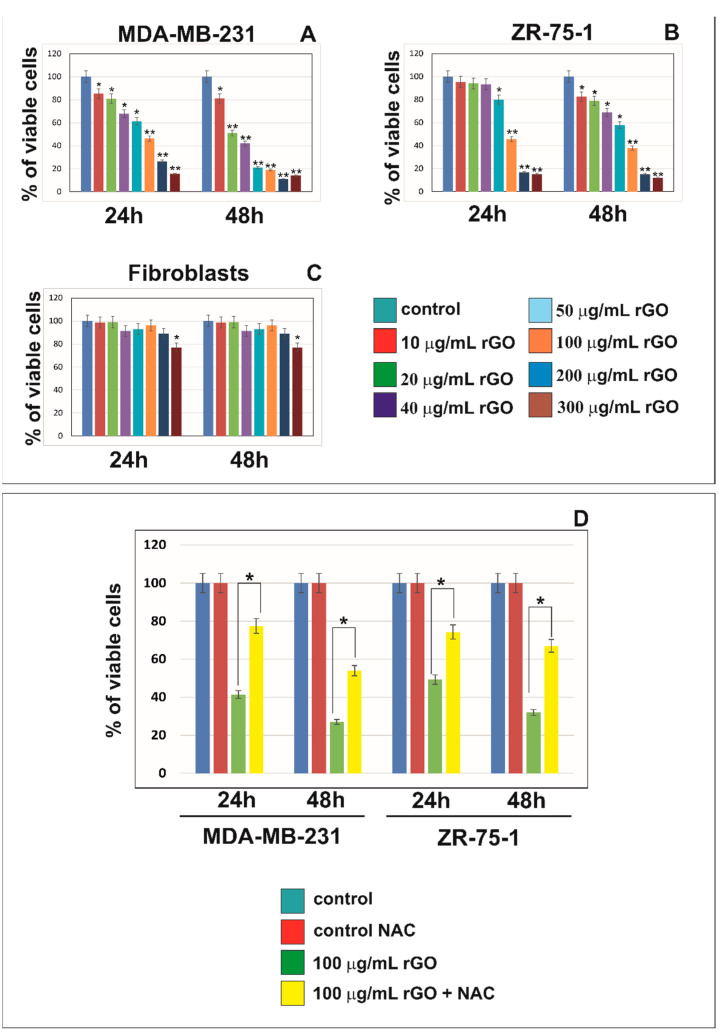
The viability of MDA-MB-231 (**A**), ZR-75-1 (**B**) and human skin fibroblasts (**C**) treated with different concentrations (10 to 300 μg/mL) of rGO for 24 and 48 h. Panel (**D**) presents the viability of MDA-MB-231 and ZR-75-1 cells treated with 100 μg/mL of rGO or 100 μg/mL of rGO + NAC (5 mmol/L), in comparison to the control cells, for 24 and 48 h. Mean values from three independent experiments ± SD are presented. Significant alterations are expressed relative to controls and marked with asterisks. * *p* < 0.05 or ** *p* < 0.001.

Figure 1 shows the viability of MDA-MB-231 and ZR-75-1 cells treated with different concentrations (10 to 300 μg/mL) of rGO for 24 and 48 h. The MTT assay performed using MDA-MB-231 and ZR-75-1 cells demonstrated that rGO reduced cell survival (Figure 1A,B). It has been shown that rGO caused the time- and dose-dependent reduction of cell viability in both MDA-MB-231 and ZR-75-1 cell lines. The decrease in cell the viability of MDA-MB-231 and ZR-75-1 cells was observed after 24 h. In cells treated with higher concentrations of rGO, the effect on cell viability was markedly more pronounced. After 48 h of incubation with rGO, MDA-MB-231 cells exhibited a greater decrease in cell viability compared to ZR-75-1 cells. In contrast to MDA-MB-231 and ZR-75-1 cell lines, normal skin fibroblasts showed a slight reduction in viability after incubation with 300 μg/mL rGO (Figure 1C).

In our previous study, we showed that rGO induced intracellular reactive oxygen species (ROS) generation in both MDA-MB-231 and ZR-75-1 breast cancer cell lines. The ROS generation concurrently occurred with the cytotoxic effects of rGO on MDA-MB-231 and ZR-75-1 cells [21]. In the present study, the effect of the antioxidant NAC (5 mmol/L) on the viability of MDA-MB-231 and ZR-75-1 cells was assessed. NAC significantly increased MDA-MB-231 and ZR-75-1 cell viability after incubation with 100 μg/mL of rGO + NAC in comparison to the cells incubated only with 100 μg/mL of rGO after 24 and 48 h (Figure 1D). 

### 2.2. rGO Induces S Phase Arrest in Breast Cancer Cells

**Figure 2:**
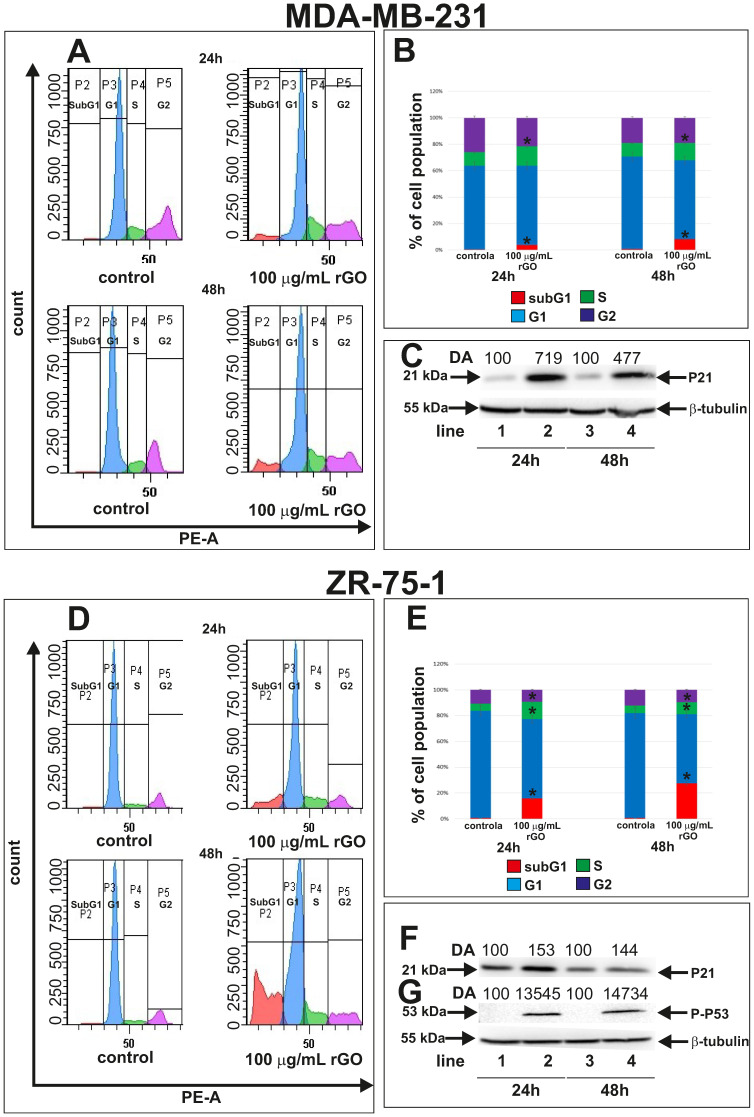
Flow cytometric analysis of MDA-MB-231 (**A**) and ZR-75-1 (**D**) cells treated with 100 µg/mL of rGO and later stained with propidium iodide. Histogram representation of the cell cycle profiles obtained from flow cytometry measurements (**A**,**D**) and a bar graph presenting the percentage of cell cycle distribution in MDA-MB-231 (**B**) and ZR-75-1 (**E**) cells. Western blot and densitometric analysis of P21 expression in MDA-MB-231 (**C**) and ZR-75-1 (**F**) and P-P53 expression in ZR-75-1 (**G**) cells incubated with 100 µg/mL rGO for 24 and 48 h. Samples containing 15 μg of protein were submitted to electrophoresis and immunoblotting. Densitometric analysis (DA) was presented as a relative fold change in comparison to untreated controls, expression level of which was set as 100. The expression of β-tubulin served as a control for protein loading. Mean values of flow cytometry analysis from three independent experiments ± SD are presented. * *p* < 0.05.

Figure 2 shows the flow cytometric analysis of MDA-MB-231 and ZR-75-1 cells treated with 100 µg/mL of rGO for 24 and 48 h. The inhibition of the cell cycle may be a result of the induction of apoptosis in cancer cells. We investigated the influence of rGO on cell cycle phases (subG1, G1, S, G2) in MDA-MB-231 and ZR-75-1 cells by flow cytometry. This assay enabled us to quantify the percentage of cells in the different phases of the cell cycle (Figure 2A,B,D,E). For this analysis, cells were treated with 100 µg/mL of rGO for 24 and 48 h. A greater percentage of cells were in the S phase after 48 h of rGO incubation compared to 24 h of incubation in both cell lines. Conversely, fewer cells were extant in the G1 and induced subG1 phase after 48 h of rGO incubation compared to 24 h. 

The percentage of rGO-treated MDA-MB-231 and ZR-75-12 cells in the S phase was significantly higher than the untreated control cells; whereas the percentage of rGO-treated cells in the G1 phase was significantly lower than control cells incubated without of rGO (Figure 2B,E).

The percentage of cells in the S phase in untreated MDA-MB-231 cells was 10.3 ± 0.17% and 10.4 ± 0.7% after 24 and 48 h, respectively (Figure 2A,B). Moreover, after 24 and 48 h of incubation with rGO at 100 µg/mL, the percentage of MDA-MB-231 cells in the S phase was 14.76 ± 0.56% and 13.3 ± 0.7%, respectively. Fewer rGO-treated cells were in the G1 phase compared to control, untreated cells after 24 and 48 h. Additionally, we also observed a greater subG1 population of rGO-treated cells after 24 and 48 h of incubation compared to control cells.

ZR-75-1 cell lines showed similar results (Figure 2D,E). The percentage of cells in the S phase in ZR-75-1 was 5.4 ± 0.50% and 5.7 ± 0.51% after 24 and 48 h, respectively, for control cells. After 24 and 48 h of incubation with 100 µg/mL rGO, the percentage of cells in the S phase was 13.4 ± 1.01% and 9.3 ± 0.70%, respectively. The number of cells in the G1 phase decreased after 48 h compared to 24 h. We also observed a greater percentage of rGO-treated cells in the subG1 phase after 24 and 48 h of incubation compared to control, untreated cells.

To validate these results, we carried out a Western blot analysis of P21 and P-P53, which are involved in the inhibition cell cycle progression (Figure 2C,F,G). We observed increased P21 expression in both MDA-MB-231 and ZR-75-1 cells incubated with 100 µg/mL of rGO (Figure 2C,F). Similarly, P-P53 expression in ZR-75-1 cells incubated with 100 µg/mL of rGO was higher compared to the untreated control cells (Figure 2G). Next, we conducted densitometry analysis and calculated the expression of P21 and P-P53 (Figure 2C,F,G). 

### 2.3. rGO Induced Apoptosis in MDA-MB-231 and ZR-75-1 Cells

**Figure 3:**
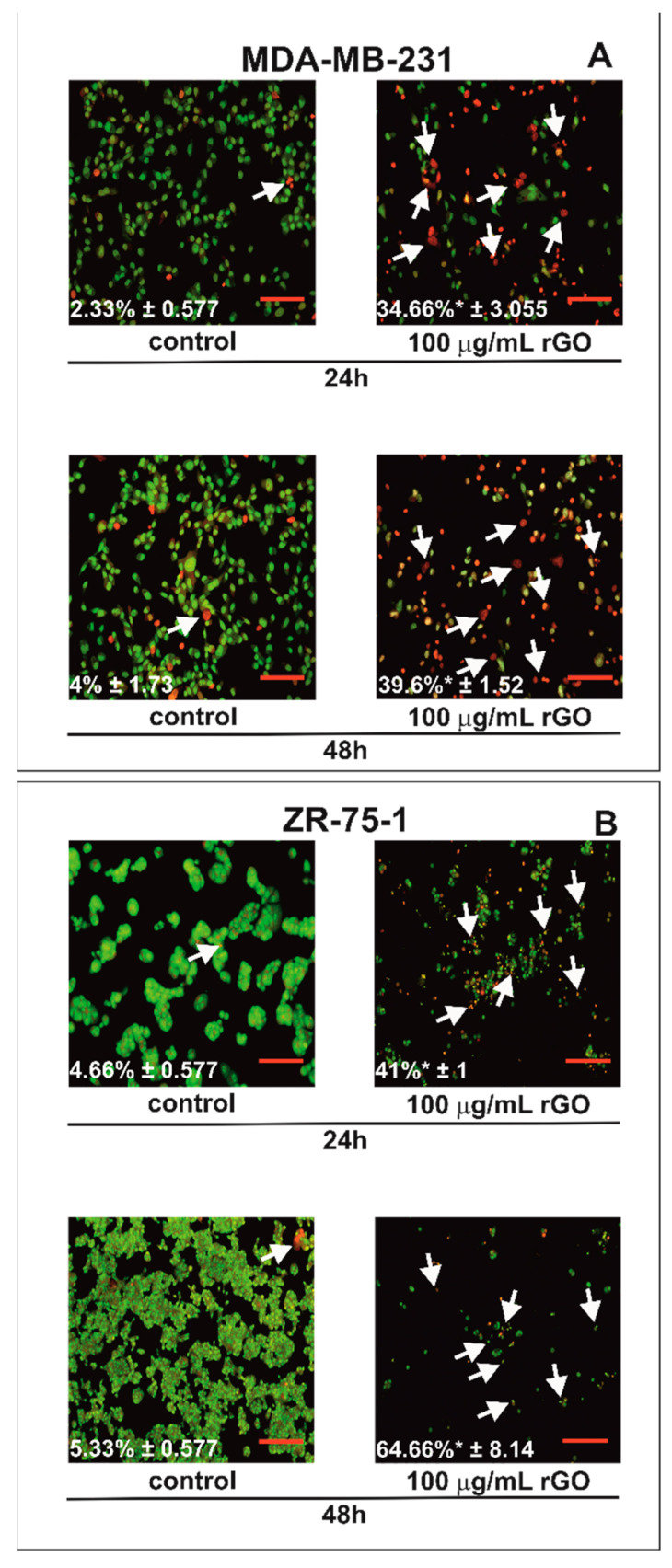
The effect of rGO on apoptosis in MDA-MB-231 (**A**) and ZR-75-1 (**B**) cells evaluated using fluorescence microscopy. The cells were incubated with 100 µg/mL of rGO for 24 and 48 h and stained with acridine orange and ethidium bromide. The cells were imaged using fluorescence microscopy at 100-fold magnification and analyzed to identify living and apoptotic cells. The percentage of apoptotic cells is shown in the lower left corner of the images. White arrows indicate apoptotic cells. We presented representative images from one of three independent experiments. Mean values from three independent experiments ± SD are presented. * *p* < 0.05. Scale bar is 50 µm.

Fluorescence microscopy was used to the evaluate apoptotic cell morphology (Figure 3). By staining with acridine orange and ethidium bromide, viable cells were distinguished by their green nuclei without the condensation of chromatin. In contrast, apoptotic cells exhibited green or red nuclei with the condensation or fragmentation of chromatin. MDA-MB-231 and ZR-75-1 cells were treated with 100 µg/mL of rGO for 24 and 48 h. rGO induced apoptosis in time-dependent manner (Figure 3A,B). The percentage of apoptotic cells is shown in the lower left corner of the photos. We observed significant changes in nuclear morphology between rGO-treated cells compared to untreated control cells after 24 and 48 h. After 24 h of rGO incubation, we noticed the apoptotic cells had condensed, fragmented and marginalized chromatin and nuclear shrinking compared to control cells in both cell lines that were investigated. Furthermore, apoptotic bodies and the shrinkage of the cells were also observed. The nuclei of cells incubated for 48 h with rGO at 100 µg/mL were redder with condensed chromatin, indicative of apoptosis. Additionally, after 48 h of rGO-treatment, there was increased cell shrinkage and a greater number of apoptotic bodies in both cell lines. 

### 2.4. The Effect of rGO on Mitochondria Dysfunction in MDA-MB-231 and ZR-75-1 Cells

In order to evaluate the effect of rGO treatment on the permeabilization of the mitochondrial membrane, the assessment of the changes in mitochondrial membrane potential (ΔΨ_m_) was performed.

**Figure 4:**
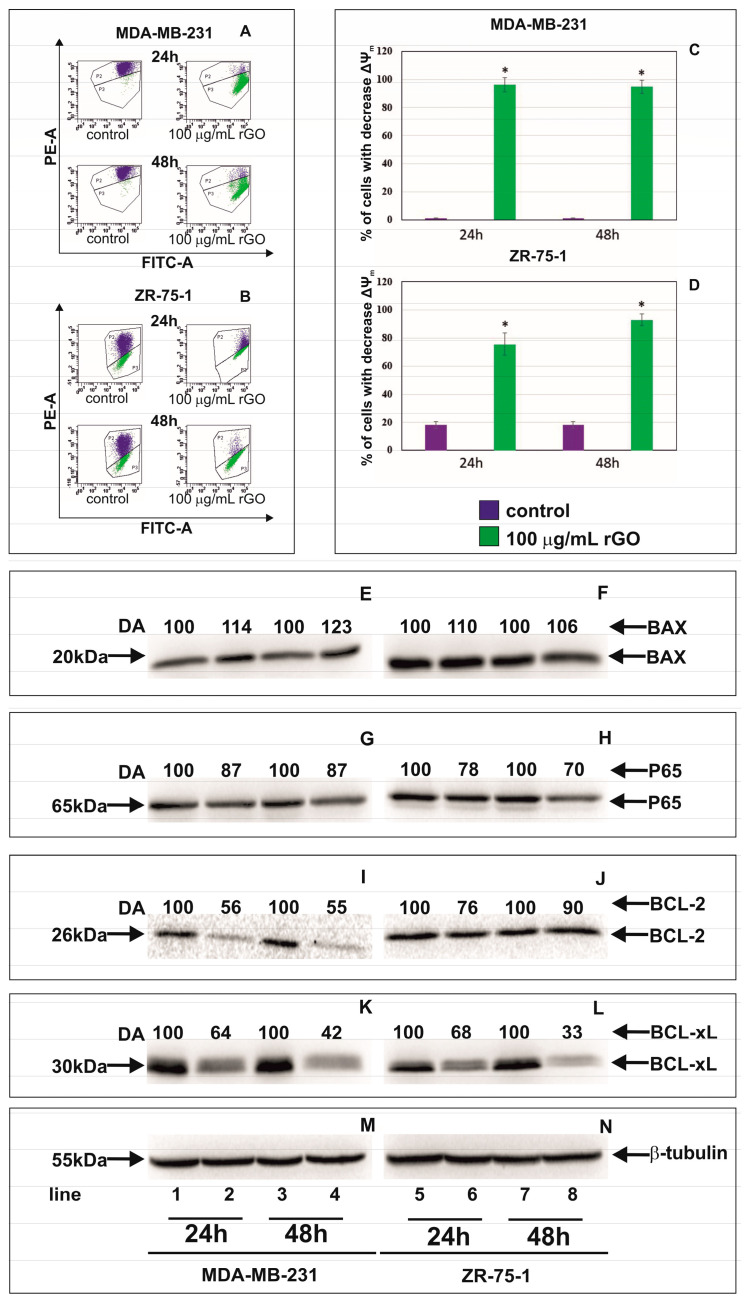
The effect of rGO on mitochondria dysfunction in MDA-MB-231 and ZR-75-1 cells. Cells were treated with 100 μg/mL of rGO for 24 and 48 h. Panels (**A**,**B**) show the flow cytometry analysis of ΔΨ_m_ in MDA-MB-231 (**A**) and ZR-75-1 (**B**) cells. X-axis and Y-axis are green and red fluorescence, respectively. Gate P2—populations of cells with normal ΔΨ_m_; gate P3—population of cells with decreased ΔΨ_m_. The right panels (**C**,**D**) show the percentage of MDA-MB-231 (**C**) and ZR-75-1 (**D**) cells with decreased ΔΨ_m_. Panels E-L show the Western blot and densitometric analysis of BAX (**E**,**F**), P65 (**G**,**H**), BCL-2 (**I**,**J**) and BCL-xl (**K**,**L**) expression in MDA-MB-231 (**E**,**G**,**I**,**K**) and ZR-75-1 (**F**,**H**,**J**,**L**) cells incubated with 100 μg/mL of rGO (lines 2, 4, 6 and 8), in comparison to the control cells (lines 1, 3, 6 and 7), for 24 and 48 h. Samples containing 15 μg of protein were submitted to electrophoresis and immunoblotting. A representative Western blot is presented. Densitometric analysis was presented as a relative fold change in comparison to untreated controls, where expression level was set as 100. The expression of β-tubulin (**M**,**N**) served as a control for protein loading. The mean values of densitometric analysis from three independent experiments ±SD are presented. * *p* < 0.001.

Figure 4A–D shows the effect of rGO on mitochondrial membrane potential (ΔΨ_m_) in MDA-MB-231 and ZR-75-1 cells. We observed a reduction in mitochondrial membrane potential in both cell lines. After 24 and 48 h of incubation, a significant loss in ΔΨ_m_ was observed. The MDA-MB-231 and ZR-75-1 cells treated with 100 µg/mL of rGO showed an approximately 90-fold and 4-fold decrease in ΔΨ_m_, respectively, in comparison to the control cells, after 24 and 48 h of incubation.

### 2.5. The Effect of rGO on Apoptosis Markers

In order to evaluate the effect of rGO treatment on the apoptosis Western blot analysis of apoptosis marker expression was performed. Figure 4E–L shows Western blot analysis of apoptosis marker expression in MDA-MB-231 and ZR-75-1 cells incubated with 100 μg/mL of rGO for 24 and 48 h. The protein expression of BAX, P65, BCL-2 and BCL-xL in MDA-MB-231 and ZR-75-1 cells exposed to rGO at concentrations of 100 μg/mL for 24 and 48 h was measured. Our results showed that the expression of BAX increased and P65, BCL-2 and BCL-xL decreased in MDA-MB-231 and ZR-75-1cells incubated with rGO compared to control cells. Next, we conducted densitometry analysis and calculated expression of BAX, P65, BCL-2 and BCL-xL. These results indicated that rGO induces apoptosis by increasing the proapoptotic protein BAX and decreasing the antiapoptotic proteins BCL-2, BCL-xL and P65 in tested human breast cancer cells.

**Figure 5:**
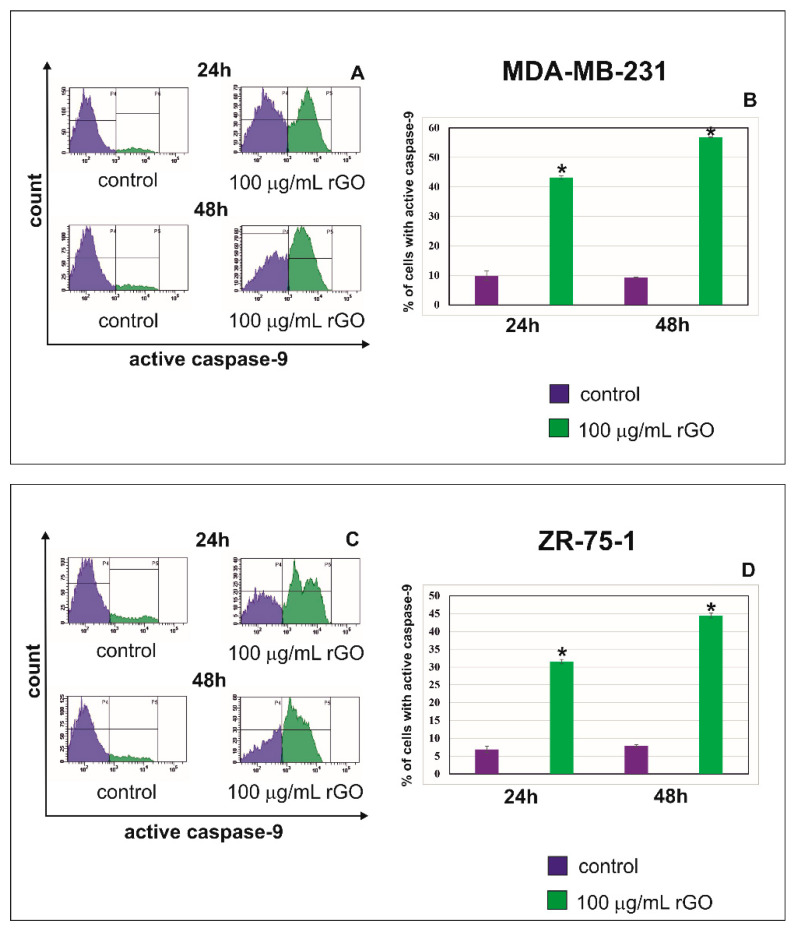
The flow cytometry analysis of active caspase-9 in MDA-MB-231 (**A**,**B**) and ZR-75-1 (**C**,**D**) cells. The cells were treated with 100 μg/mL of rGO for 24 and 48 h. Panels (**A**,**C**) show the representative histograms of MDA-MB-231 (**A**) and ZR-75-1 (**C**) cells stained with FAM-FLICA Caspase-9. Panels (**B**,**D**) show the percentage of MDA-MB-231 (**B**) and ZR-75-1 (**D**) cells with active caspase-9. Gate P4—populations of cells without of active caspase-9; gate P5—population of cells with active caspase-9. The right panel shows the percentage of MDA-MB-231 (**B**) and ZR-75-1 (**D**) cells with active caspase-9. Significant alterations are expressed relative to controls and marked with asterisks. * *p* < 0.001.

**Figure 6:**
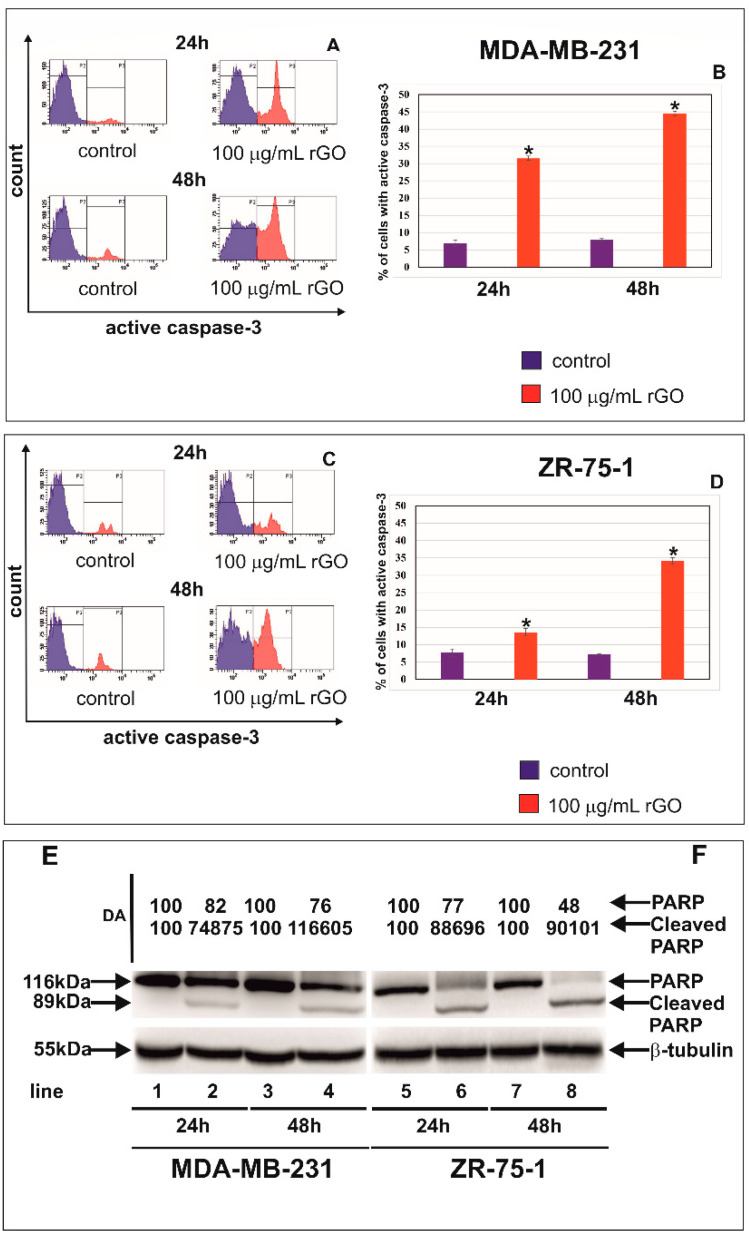
The effect of rGO on active caspase-3, PARP and cleaved-PARP expression in MDA-MB-231 and ZR-75-1 cells. The cells were treated with 100 μg/mL of rGO for 24 and 48 h. Panels (**A**,**B**) show the flow cytometry analysis of active caspase-3 in MDA-MB-231 (**A**,**B**) and ZR-75-1 (**C**,**D**) cells. Panels (**A**,**C**) show the representative histograms of MDA-MB-231 (**A**) and ZR-75-1 (**C**) cells. Panels (**B**,**D**) show the percentage of MDA-MB-231 (**B**) and ZR-75-1 (**D**) cells with active caspase-3. Gate P2—populations of cells without of active caspase-3; gate P3—population of cells with active caspase-3. The right panel shows the percentage of MDA-MB-231 (**B**) and ZR-75-1 (**D**) cells with active caspase-3. The Western blot analysis of PARP and cleaved-PARP was performed in MDA-MB-231 (**E**) and ZR-75-1 cells (**F**) for rGO-treated cells (lines 2, 4, 6, and 8) and untreated control cells (lines 1, 3, 5, and 7). Samples containing 15 μg of protein were submitted to electrophoresis and immunoblotting. Densitometric analysis (**E**,**F**) was presented as a relative fold change in comparison to untreated controls, where the expression level was set as 100. The expression of β-tubulin served as a control for protein loading. The mean values of analysis from three independent experiments ± SD are presented. * *p* < 0.05. Significant alterations are expressed relative to controls and marked with asterisks.

The cysteine aspartyl protease, caspase-9, plays an important role in the mitochondrial or intrinsic apoptotic pathway, which is engaged in the response to many apoptotic stimuli. Once activated, caspase-9 cleaves and activates the effector caspase-3 to bring about apoptosis [24]. Figure 5A–D shows the effect of rGO on caspase-9 activated in MDA-MB-231 (A,B) and ZR-75-1 (C,D) cells. Figure 6A–D shows the effect of rGO on active caspase-3 in MDA-MB-231 (A,B) and ZR-75-1 (C,D) cells.

In the current study, the effect of rGO treatment on the activation of caspase-9 (Figure 5) and caspase-3 (Figure 6) was investigated after 24 and 48 h of incubation. We observed a time-dependent increase of active caspase-9 and active caspase 3 after treatment with 100 µg/mL rGO in comparison to control cells in both MDA-MB-231 and ZR-75-1 cell lines. The highest percentage of MDA-MB-231 and ZR-75-1 cells with active caspase-9 and active caspase 3 was observed after 48 h in comparison to control cells (Figure 5 and Figure 6). 

To validate these results, we carried out the Western blot analysis of PARP, which is involved in apoptosis (Figure 6E,F). We observed a decrease in PARP expression and the increased expression levels of cleaved-PARP in cells incubated with 100 μg/mL of rGO in comparison to control cells in both MDA-MB-231 and ZR75-1 cell lines. Next, we conducted densitometry analysis and calculated the expression levels of PARP and cleaved-PARP (Figure 6E,F). 

### 2.6. The Effect of rGO on Autophagy Markers

**Figure 7:**
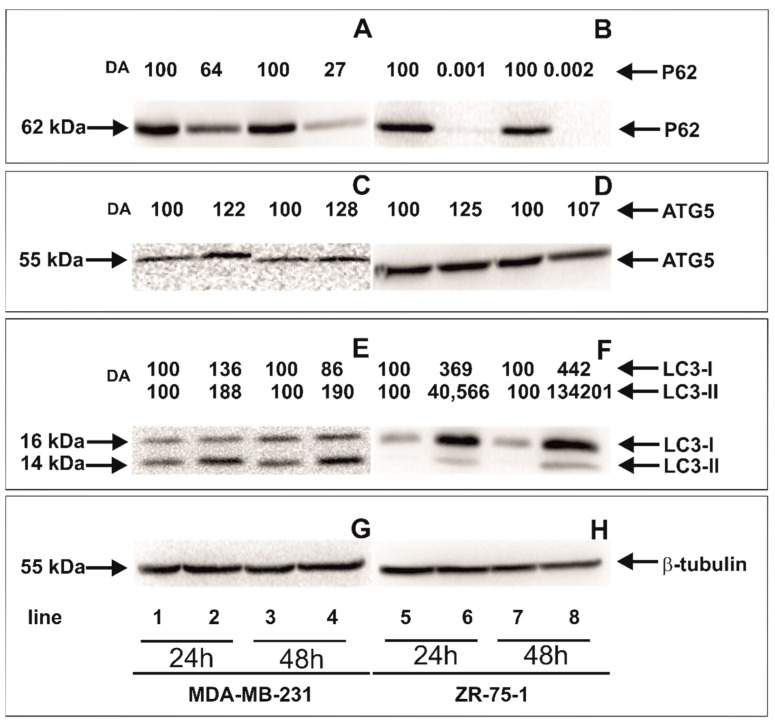
Western blot and densitometric analysis of P62 (**A**,**B**), ATG5 (**C**,**D**), LC3-I and LC3-II (**E**,**F**) expression in MDA-MB-231 (**A**,**C**,**E**) and ZR-75-1 (**B**,**D**,**F**) cells incubated with 100 μg/mL of rGO (lines 2, 4, 6 and 8) in comparison to untreated control cells (lines 1, 3, 5 and 7) for 24 (lines 1, 2, 5 and 6) and 48 h (lines 3, 4, 7, and 8). Samples containing 15 μg of protein were submitted to electrophoresis and immunoblotting. A representative Western blot is presented. Densitometric analysis was presented as a relative fold change in comparison to untreated controls, where expression level was set as 100. The expression of β-tubulin (**G**,**H**) served as a control for protein loading.

Figure 7 shows the expression levels of autophagy markers P62, ATG5, LC3 I and LC3-II in MDA-MB-231 and ZR-75-1 cells incubated with 100 μg/mL of rGO in comparison to control, untreated cells for 24 and 48 h determined using Western blot analysis. After 24 and 48 h incubation with 100 μg/mL of rGO both cell lines expressed LC3-I and LC3-II. Moreover, we observed that the expression of membrane-bound LC3-II was increased in cells incubated with 100 μg/mL of rGO after 24 and 48 h in comparison to the untreated, control MDA-MB-231 and ZR-75-1 cells. Our results also showed that the expression of P62 decreased and ATG5 increased in MDA-MB-231 and ZR-75-1 cells incubated with 100 μg/mL of rGO in comparison to control cells. 

## 3. Discussion

Breast cancer cell lines have been applied widely to investigate breast cancer pathobiology and to screen and characterize new nanotherapeutics. The MDA-MB-231 and ZR-75-1 cell lines are widely applied in breast cancer in vitro modeling [22,25]. The mechanisms of rGO nanoparticle cytotoxicity are not completely understood and depend on their physiochemical properties [22]. One of the main causes of rGO cytotoxicity are physical interactions of rGO with the cell membrane. Theproperties of rGO play a critical role in graphene distribution and toxicity, leading to a decline in cell viability [22]. Additionally, the strong hydrophobic interactions of rGO with the cell membrane can lead to cytoskeletal dysfunction. Interestingly, the sharpened edges of rGO may act as ‘blades’, inserting and damageing cell lipids [26]. 

The cytotoxicity of MDA-MB-231 and ZR-75-1 cells exposed to rGO was studied using the MTT assay. rGO caused time- and dose-dependent reductions in cell viability in both MDA-MB-231 and ZR-75-1 cell lines. In contrast to the MDA-MB-231 and ZR-75-1 cell lines, only a modest reduction in cell viability was observed when fibroblasts cells were treated with the highest concentration of rGO investigated (300 µg/mL). The differences in the sensitivity of breast cancer cell lines to rGO in comparison to the fibroblasts cells may result from differences in the expression of genes involved in apoptosis or autophagy. rGO have the potential to be used as anticancer drugsin breast cancer therapy [22]. Gurunathan et al. used mushroom extracts for GO reduction. The cytotoxicity of graphene was dose-dependent manner [27]. Mendes et al. indicated that larger graphene flakes reduce cell viability when equate to smaller flakes [28]. Jing et al. indicated that GO (≥100 µg/mL) exhibited time- and dose-dependent cytotoxicity against breast cancer cell line [29]. Alsaedi et al. demonstrated that rGO-treatment caused a significant and concentration-dependent decrease in the growth of MCF-7 in comparison to untreated control cells [30]. rGO also exhibited toxicity against glioblastoma U87, U-118 MG, U251 cell lines [31,32,33]. Kang et al. researched PC12 cell line using the CCK-8 test, which revealed that GO and rGO led to decreases in the cells’ viability [34]. 

GO can break the integrity of the cells by manipulating the expression of membrane- and cytoskeleton-associated genes. After internalization, GO might be localized on F-actin filaments and thus induce cell cycle arrest, apoptosis or oxidative stress [35]. Oxidative stress is defined as an imbalance among the production of ROS and their elimination by antioxidants. The ROS are produced in the cells during the the functioning of the mitochondrial respiratory chain... During endogenous metabolic reactions, ROS, such as superoxide anion (O^2-^), hydrogen peroxide (H_2_O_2_) and hydroxyl radical (OH^•^), which play pivotal roles in cell metabolism [36]. Cancer cells exhibit aberrant redox homeostasis; but while ROS are pro-tumorigenic, high ROS levels are cytotoxic [36]. In our previously study, we showed that rGO induced intracellular ROS generation in both MDA-MB-231 and ZR-75-1 breast cancer cell lines [22]. 

To analyze whether ROS generation was the reason for decreased cell viability in rGO-treated MDA-MB-231 and ZR-75-1 cells, an ROS scavenger, NAC, was also added to the cells with and without rGO-treatment. NAC is a precursor to the major antioxidant glutathione. The mechanisms responsible for the beneficial effects of NAC have been associated partly with its antioxidant properties [36,37]. In our study, NAC (5 mmol/L) significantly increased the cell viability of rGO-treated cells in comparison to the cells incubated only with 100 µg/mL rGO (no NAC) in breast cancer MDA-MB-231 and ZR-75-1 cells. This process may indicate a direct cytotoxic effect of ROS, induced by rGO, on breast cancer cells. A similar effect was observed by Tang et al. [38]. The investigators indicated that a NAC could alleviate the cytotoxic effect of GO in K_7_M_2_ cell line [38]. 

Increases in ROS production could lead to the mitochondrial damage and perturbation in the mitochondrial membrane potential (ΔΨ_m_) of cancer cells [36]. In our study we observed apoptosis as well as reduction in mitochondrial membrane potential in MDA-MB-231 and ZR-75-1 cells cultured with rGO. Apoptosisis a process leading to cell death characterized by cell shrinkage, membrane blebbing, DNA fragmentation and making of apoptotic bodies. There are two main apoptotic pathways: exogenous (death receptor) pathway and intrinsic (mitochondrial) pathway [39]. Due to the low mitochondrial membrane potential (MMP) and changes in apoptotic cell morphology, we suggest that rGO-induced apoptosis in MDA-MB-231 and ZR-75-1 cells occurs through the mitochondrial pathway. Mitochondria play a crucial role in the activation of the mitochondrial pathway of apoptosis in cancer cells. Lammel et al. indicated that incubating cells with GO results in the disruption of the mitochondrial membrane [40]. This phenomenon was also associated with a decrease in the MMP [41,42]. 

The mechanism of apoptosis induced by different nanomaterials relies on a decrease in BCL-2 antiapoptotic protein expression and an increase in the synthesis of the proapoptotic proteins, such as BIM and BAX. In our study, we showed that rGO led to increased BAX and decreased BCL-xL and BCL-2 level expression. The BIM protein increases apoptosis on the way binding and neutralizing certain antiapoptotic proteins. [43]. The Bax protein contain α-helical domains, which participate in the formation of channels in mitochondrial membrane. BAX protein connects with permeability transition pores, and increases the permeability of the mitochondrial membrane for cytochrome c [43]. Apaf-1 complex with cytochrome c activates procaspase-9. The caspase-9 enacts an important role in the mitochondrial or intrinsic apoptotic pathway that is engaged in answer to many apoptotic stimuli [43]. Active caspase 9 leads to the activation of executive caspase-3 [39]. PARP activates caspases-3 and -7 in caspase-dependent apoptosis. In our study we showed a time-dependent increase in active caspase-9, active caspase-3 and the increased expression of cleaved-PARP (89 kDa) in MDA-MB-231 and ZR-75-1 cells. Nadi et al. indicated that the downregulation of PARP (116 kDa) expression as compared to untreated cells can be attributed to its cleavage because of DNA damage [43]. Jin-Huan et al. found that after 48 h treatment with Ori@GE11-GO, the expression of the antiapoptotic proteins was clearly decreased in KYSE-30 and EC109 cells. The expression levels of the proapoptotic proteins showed no significant alter in KYSE- 30 cells but showed increased expression levels in EC109 cells. These results further supported that Ori@GE11-GO treatment significantly induced the apoptosis of KYSE-30 and EC109 cells. Hence, the activation of the intrinsic apoptotic pathway is also associated with Ori@GE11-GO [44]. Thus, the expression of BAX and the cleavage of PARP were used to assess the apoptotic tendency of the investigated MDA-MB-231 and ZR-75-1 cells.

Nuclear factor NF-κB is a protein that has a role in adjusting DNA transcription and is considered an apoptosis inhibitor [45]. The expression of the NF-κB P65 subunit has been shown to advertise cell proliferation, whereas the inhibition of NF-κB activation blocks cell proliferation. In our previous study, we demonstrated that rGO caused time- and dose-dependent decreases in proliferation in MDA-MB-231 and ZR-75-1 cells [22]. The mechanism for the activation of NF-κB by ROS is not clear, and the relationship between NF-κB and ROS is complex [36]. In our study, we observed that rGO decreased the expression of the NF-κB P65 subunit in MDA-MB-231 and ZR-75-1 cells in a time-dependent manner, and this correlates with the induction of apoptosis. This finding suggests that rGO at 100 µg/mL can induce apoptosis by suppressing P65 in breast cancer cells.

We further examined the effect of rGO on cell cycle distribution in MDA-MB-231 and ZR-75-1 after 24 and 48 h of incubation. In our study, we showed that rGO treatment increased the percentage of cells in the S phase decreased the percentage of cells in the G1 phase and induced the subG1 phase in both cell lines. Furthermore, to validate these results, we conducted Western blot analyses of P21 and P53 proteins that are concerned in the inhibition of cell cycle progression. The cell cycle is composed of four phases and regulated by the activation and inactivation of cyclin-dependent kinases depending upon the situations of the cell and the surrounding environment. The dysregulation of the cell cycle leads to cancerous transformation and therefore serves as a potential target for cancer therapeutics [46]. It has been assumed that the cell cycle inhibition of S phase is mediated by the induction of P21 and P53 [47]. We noticed a time-dependent increase of P21 expression in tested breast cancer cells and increased P-P53 expression in ZR-75-1 cells treated with 100 µg/mL of rGO in comparison to untreated control cells after 24 and 48 h of incubation The Cdk inhibitor P21 may be induced by both P53-dependent and P53-independent mechanisms, and the induction of P21 is attributable to cell cycle arrest [48]. The upregulation of P53 and P21 in ZR-75-1 cells and P21 in MDA-MB-231 cells indicated cell cycle arrest. There is reported that mutant P53 is involved in the survival of MDA-MB-231 [48]. In MDA-MB-231 cells, P21 expression patterns can be regulated by the P53-independent pathway [49]. Wang et al. showed that GO arrested the cell cycle in S phase [50]. Hashemi et al. revealed that GO particles increased DNA synthesis by ROS generation and damaging the DNA of fibroblasts [50]. This may indicate that the S cell cycle arrest observed in our study can be mediated by P21.

The number of studies on rGO-induced autophagy and autophagy cell death has increased in recent years. Autophagy is an evolutionary catabolic process, and it plays an pivotal role in cell survival pending the stress conditions [51]. Paradoxically, this process can buffer cancer cells but may also contribute to cell death if the stress is prolonged [52]. Autophagy is controlled via ATG proteins. For example, ATG5 can lead to the induction, activation and nucleation of autophagic vesicles [53]. During autophagy, double membrane autophagosomes mass in order to engulf intracellular components. The cytosolic form of LC3-I is conjugated with phosphatidylethanolamine during the process of autophagosomal membrane formation and subsequently generates LC3-II [51]. The conversion of LC3-I into LC3-II is generally used to monitor autophagy and is given as the biomarker of this process [50]. Since P62 accumulates when autophagy is inhibited, and decreased levels can be observed when autophagy is induced, P62 may also be used as a marker of this proces [53]. In the present study, the expression levels of autophagy-related proteins (ATG5, P62, LC3-I and LC3-II) were assessed to warrant the role of autophagy in breast cancer cells incubated with rGO. The results showed that rGO (100 µg/mL) increased the level of ATG5 and LC3-II expression and decreased the level of P62 expression in MDA-MB-231 and ZR-75-1 cells. There is a near association between oxidative stress and autophagy. ROS might induce autophagy by associated signaling pathway proteins, such as mTOR [46]. mTOR activity is impacted by multiple signaling pathways, such as AMPK and PI3K/AKT. Vice versa, autophagy activation advertise the production of catalase by the degradation of selective autophagic inhibitors, leading to ROS accumulation, and ultimately resulting in a positive feedback regulatory loop between autophagy and ROS [53]. Furthermore, we noticed the coexistence of autophagy and apoptosis in MDA-MB 231 and ZR-75-1 cells exposed to rGO. Latterly, a few research indicated that autophagy increased significantly after exposure to rGO. Tang et al. indicated that autophagy was simultaneously stimulated by characteristic autophagosome formation, autophagy flux, and increased the expression level of autophagy-related proteins (LC3-I to LC3-II conversion, ATG5 and ATG7) [38]. ATG5 is one of the components of the basic autophagic machinery. The key point in this mechanism is the cleavage of ATG5 by calpain to create a cut form of the protein that translocates into the mitochondria membrane, opens the mitochondrial permeability transition pores (MPTP) and activates the intrinsic apoptosis pathway [51].

**Figure 8:**
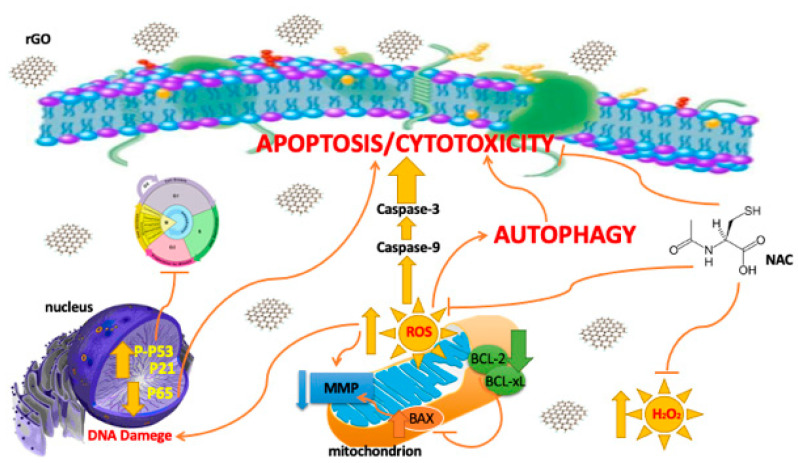
The effect of rGO on cytotoxicity, apoptosis, autophagy, oxidative stress and cell cycle in breast cancer cells. Abbreviation: BAX— Bcl-2-associated X protein; BCL-2—B-cell CLL/lymphoma 2; BCL-xL—B-cell lymphoma-extra-large; MMP—mitochondrial membrane potential; NAC—N-acetylcysteine; rGO—reduced graphene oxide; ROS—reactive oxygen species.

Our results suggest that rGO can enhance cytotoxicity in breast cancer MDA-MB-231 and ZR-75-1 cells but fibroblasts cells. Interestingly, we observed the coexistence of apoptosis and autophagy in breast cancer cells (Figure 8).

## 4. Materials and Methods

### 4.1. Reagents

L-15 Medium (1×) + GlutaMAX^TM^-I, trypsin-EDTA, RPMI Medium 1640 (1×) + GlutaMAX^TM^-I, DPBS, penicillin, strptomycin, and fetal bovine serum Gold (FBS Gold) were provided by Gibco (San Diego, CA, USA). JC-1 MitoScreen Kit and PE Active Caspase-3 Apoptosis Kit were obtained from BD Pharmingen^TM^ (San Diego, CA, USA). Reduced graphene oxide (rGO), acridine orange, ethidium bromide, N-acetylcysteine and propidium iodide were obtained from Sigma-Aldrich (St. Louis, MO, USA). BCA Protein Assay Kit and Pierce^TM^ ECL Western Blotting Substrate were obtained from Thermo Scientific (Rockford, IL, USA). RIPA buffer and Protease/Phosphatase Inhibitor Cocktail were provided by Cell Signalling Technology (Boston, MA, USA) and the BCA Protein Assay Kit was provided by Thermo Scientific (Rockford, IL, USA). The FAM-FLICA Caspase-9 Assay was obtained from ImmunoChemistry Technologies (Davis, CA, USA). Immuno-Blot PVDF Membranes for Protein Blotting were obtained from Bio-Rad (Hercules, CA, USA). Monoclonal (mouse) anti-human SQSTM1/P62 antibody, monoclonal (rabbit) anti-human NF-kB P65 antibody, monoclonal (rabbit) anti-human P21 Waf1/Cip1 antibody, monoclonal (rabbit) anti-human BCL-xL antibody, monoclonal (rabbit) anti-human BCL-2 antibody, monoclonal (rabbit) anti-human BAX antibody, monoclonal (rabbit) anti-human Phospho-P53 antibody, monoclonal (rabbit) anti-human ATG5 antibody, polyclonal (rabbit) anti-human LC3A/B, monoclonal (rabbit) anti-human poly(ADP-ribose) polymerase (PARP) antibody, polyclonal (rabbit) anti-human β-tubulin antibody, anti-rabbit IgG, horseradish peroxidase (HRP) antibody and anti-mouse IgG, HRP antibody were obtained from Cell Signaling Technology (Boston, MA, USA).

### 4.2. Cell Cultures and Exposure to rGO 

Human breast cancer cell lines MDA-MB-231 and ZR-75-1 as well as human skin fibroblasts (CRL 1474) were obtained from American Type Culture Collection (ATCC). MDA-MB-231 cells were cultured in L-15 Medium (1×) + GlutaMAX^TM^-I and ZR-75-1 cells were cultured in RPMI Medium 1640 (1×) + GlutaMAX^TM^-I, while the human skin fibroblasts were cultured in DMEM (1×) + GlutaMAX^TM^-I. The media were supplemented with 10% heat-inactivated fetal bovine serum GOLD (FBS GOLD), penicillin (100 U/mL) and streptomycin (100 µg/mL). Cells were cultured in Falcon flasks (BD Pharmingen^TM^, San Diego, CA, USA) in CO_2_ incubator Galaxy S+ (RS Biotech, Irvine, UK), at 37 °C, 5% CO_2_ (or without CO_2_-MDA-MB-231) and 95% air in an incubator Galaxy S+ (RS Biotech, Irvine, UK). At approximately 70% confluence, cells were detached with 0.05% trypsin, 0.02% EDTA and counted in a Scepter Cell Counter (Millipore, MA, USA). Then, 2.5 × 10^5^ cells per well were seeded in 2 mL of L-15 Medium (1×) + GlutaMAX^TM^-I or RPMI Medium 1640 (1×) + GlutaMAX^TM^-I in six-well plates for 24 h. Prior to the experiments, nanoparticles were dispersed in deionized water (1 mg/mL) by a sonicator (Sonopuls, Bandelin, Berlin, Germany), on ice for 45 min (160 W, 20 kHz) in order to minimize the aggregation of rGO. Next, media was removed and replaced with fresh media containing rGO suspensions, at concentrations ranging from 10 μg/mL to 300 μg/mL. The cell lines not treated with rGO served as the negative controls. Thereafter, the cells were incubated for 24 h and 48 h and retained for further analyses.

### 4.3. Cell Viability

Cell viability was measured based on the methods of Carmichael et al., using 3-(4,5-dimethylthiazol-2-yl)-2,5-diphenyltetrazolium bromide (MTT) [54]. The MDA-MB-231, ZR-75-1 and fibroblasts cells were seeded in 24-well plates at a density of 2.5 × 10^4^ per well. After 24 h, the media was removed and replaced with fresh media containing rGO at concentrations ranging from 10 to 300 µg/mL. The untreated MDA-MB-231, ZR-75-1 and fibroblasts cells served as the negative controls. The MDA-MB-231, ZR-75-1 and fibroblasts cells were washed three times with phosphate buffer saline (PBS) and then incubated with 1 mL of MTT solution (0.25 mg/mL in PBS) in 5% CO_2_ incubator at 37 °C for 4 h. The medium was removed and 1 mL of 0.1 mol/L HCl in absolute isopropanol was added. The absorbance of converted dye in living cells was measured at the wavelength of 570 nm on an Infinite M200 microplate reader (Tecan, Salzburg, Austria). The viability of MDA-MB-231 and ZR-75-1 cells as well as fibroblasts cultured with rGO was calculated as the percentage of the untreated cells. All the experiments were performed in duplicates in at least three cultures.

In a separate MTT experiment, breast cancer cells were seeded as above. Then, the cells were pre-treated (for 1 h) with NAC (5 mmol/L). After pre-treatment, the cells were incubated with or without rGO for 24 and 48 h. The viability of MDA-MB-231 and ZR-75-1 cells was measured as above and calculated as the percentage of the untreated cells. All the experiments were done in duplicates in at least three cultures.

### 4.4. Cell Cycle Analysis

The distribution of the cell cycle phases was analyzed by flow cytometry. The MDA-MB-231 and ZR-75-1 cells were seeded onto 6-well plates at a density of 2.5 × 10^5^ cells per well and treated with 100 μg/mL of rGO for 24 and 48 h. At the end of the treatment, the cells were collected using 0.25% EDTA/trypsin and washed twice in 1× DPBS. Then, 70% ethanol was used to fix the cells at −20 °C for 12 h. Next, the cells were resuspended in 1× DPBS, treated with 50 μg/mL of DNase-free RNase A (AppliChem, Darmstadt, Germany), and stained with 100 μg/mL of propidium iodide. The FACSCanto II flow cytometer was used to read the fluorescence, and the percentage of cells in each phase of the cell cycle (subG1, G1, S and G2) was calculated using the FACSDiva software (ver. 6.1.3, BD Biosciences Systems, San Diego, CA, USA).

### 4.5. Fluorescent Microscopy

Cells were stained with fluorescent dyes, including acridine orange and ethidium bromide, in order to evaluate the nuclear morphology of apoptotic cells. The MDA-MB-231 and ZR-75-1 cells grew on a cover glass with rGO (100 µg/mL) for 24 and 48 h. Thereafter, the cells were washed twice with 1× DPBS and stained with 1 mL of the dye mixture (10 μM acridine orange and 10 μM ethidium bromide in 1× DPBS) for 10 min in the dark at room temperature (RT). Next, the stained solution was removed, and the cells were washed with 1× DPBS, analyzed and imaged under a fluorescence microscope at 200× magnification. Acridine orange is a vital dye that will stain both live and dead cells, whereas ethidium bromide will stain only cells that have lost their membrane integrity. Per sample, 100 cells were analyzed by fluorescence microscopy (Olympus CXK41, U-RLFT50) according to the following criteria: living cells—normal green nucleus; early apoptotic cells—bright green nucleus with condensed or fragmentated chromatin; and late apoptotic cells—orange-stained nuclei with chromatin condensation or fragmentation. The percentage of apoptotic cells was the sum of early apoptotic and late apoptotic cell percentages. 

### 4.6. Mitochondrial Membrane Potential (∆Ψ_m_) Analysis

MDA-MB-231 and ZR-75-1 cells were seeded (2.5 × 10^5^ cells/well) and incubated in 2 mL of L-15 Medium (1×) + GlutaMAX^TM^-I or RPMI Medium 1640 (1×) + GlutaMAX^TM^-I, respectively, in 6-well plates. After 24 h, media was removed and replaced with fresh media containing rGO suspensions at concentrations of 100 μg/mL and incubated for 24 and 48 h. Then, the cells were detached and were suspended in PBS at 1 × 10^6^ cells per mL. Subsequently, the disruption of the mitochondrial membrane potential in MDA-MB-231 or ZR-75-1 cells was assessed using the MitoScreen kit (BD, San Diego, CA, USA), following the manufacturer’s instructions. Briefly, supravital cells were washed in PBS and resuspended in buffer supplemented with 10 µg/mL of the lipophilic cationic probe 5,5,6,6-tetrachloro-1,1,3,3-tetraethylbenzimidazolcarbocyanine iodide (JC-1). Then, the MDA-MB-231 or ZR-75-1 cells were incubated at 37 °C for 15 min, washed and resuspended in buffer and analyzed using flow cytometry (BD FACSCanto II, San Diego, CA, USA). The percentage of cells with disrupted mitochondrial membrane potential (MMP) was calculated using the FACSDiva software.

### 4.7. Caspase-9 Enzymatic Activity Assay

Caspase-9 activity was measured using the FAM-FLICA Caspase 9 Kit (ImmunoChemistry Technologies, Davis, CA, USA) according to the manufacturer’s instructions. The MDA-MB-231 and ZR-75-1 cells were treated with 100 µg/mL rGO for 24 and 48 h. Next, the cells were harvested and washed with cold 1× DPBS. Then, 10 µL of diluted FLICA reagent was added to 290 µL of the cell suspension. The cells were incubated at 37 °C for 60 min. After incubation, the cells were washed twice with 400 µL of buffer and centrifuged at 1000× *g* for 5 min. Analysis was performed using the BD FACSCanto II (San Diego, CA, USA) flow cytometer, and results were analyzed with FACSDiva software.

### 4.8. Caspase-3 Assay

Caspase-3 was measured using the PE Active Caspase-3 Apoptosis Kit (San Diego, CA, USA)according to the manufacturer’s instructions. The MDA-MB-231 and ZR-75-1 cells were treated with 100 µg/mL of rGO for 24 and 48 h. Next, the cells were harvested and washed with cold 1× DPBS. Then, the cells were resuspended in Cytofix/Cytoperm solution at a concentration of 1 × 10^6^ cells/0.5 mL. The cells were incubated for 20 min on ice. Pellet cells were aspirated, and the remaining cells were washed twice with Perm/Wash buffer (1×) at RT. The cells were resuspended in BD Perm/Wash^TM^ buffer (1×) plus antibody and incubated for 30 min. Washes were performed using 1 mL BD Perm/Wash^TM^ buffer (1×) before finally resuspending the cell solution in 0.5 mL BD Perm/Wash^TM^ buffer (1×). Data were acquired and evaluated with FACSDiva software.

### 4.9. Western Blot Analysis

The breast cancer cells were washed with cold PBS and solubilized in 300 μL per well of RIPA buffer enriched with a protease-/phosphatase-inhibitor cocktail (100×). The lysates from each well were centrifuged at 10,000× *g*, at 4 °C, for 15 min. The samples of lysates containing 15 μg of protein were subjected to SDS-PAGE, as described by Laemmli [55]. An amount of 10% or 12% polyacrylamide gel and constant 25 mA current were used.

The proteins were transferred to PVDF membranes for protein blotting and subsequently pre-treated with Tris-buffered saline (TBS) at RT for 1 h. Each strip was treated with blocking buffer (5% non-fat dry milk in TBS containing 0.05% Tween 20 (TBS-T), pH 7.6 for 1 h. The membranes were washed once for 15 min and 4 times for 5 min in TBST. Then, the membranes were probed with the following antibodies at the indicated concentrations: monoclonal (mouse) anti-human SQSTM1/P62 antibody (1:1000), monoclonal (rabbit) anti-human NF-kB P65 antibody (1:1000), monoclonal (rabbit) anti-human P21 Waf1/Cip1 antibody (1:1000), monoclonal (rabbit) anti-human BCL-xL antibody (1:1000), monoclonal (rabbit) anti-human BCL-2 antibody (1:1000), monoclonal (rabbit) anti-human BAX antibody (1:1000), monoclonal (rabbit) anti-human Phospho-P53 antibody (1:1000), monoclonal (rabbit) anti-human ATG5 antibody (1:1000), monoclonal (rabbit) anti-human LC3A/B, monoclonal (rabbit) anti-human PARP antibody (1:1000) and polyclonal (rabbit) anti-human β-tubulin antibody (1:1000) in TBS with 5% non-fat dry milk at 4 °C for 16 h. After washing once with TBS-T for 15 min and 2 times for 5 min, each strip was incubated with HRP conjugated with anti-human secondary antibody against rabbit or mouse IgG (whole molecule) at 1:1000 dilution in TBS, under slow shaking for 2 h. The membranes were washed once for 15 min and 4 times for 5 min with TBS-T and exposed to the ECL reagent in the dark for chemiluminescence detection.

### 4.10. Chemiluminescence Detection

For chemiluminescence detection, Western blot membranes were incubated in the dark with ECL Reagent (Cell Signaling), which is a luminol-based enhanced chemiluminescence substrate for peroxidase. The luminescent signal was recorded and quantified with the GeneGnome (Syngene, Frederick, MD, USA). The luminescent signals were detected and transmitted to the GeneTools software (Syngene, Frederick, MD, USA) for analysis and documentation.

### 4.11. Total Protein Content in Cells

Protein concentration in cell lysates was measured using BCA Protein Assay Kit (Rockford, IL, USA) [56]. Bovine serum albumin was used as a standard. 

### 4.12. Statistical Analysis

Mean values from three independent experiments ± standard deviations (SD) were calculated. The data were statistically analyzed using one way-ANOVA followed by Tukey’s post hoc *t*-test analysis. The significant differences of means were determined at the level of * *p* < 0.05 or ** *p* < 0.001.

## 5. Conclusions

Due to the limited toxicological information of rGO, we examined the cytotoxic effect of rGO-treatment on breast cancer cells. In sum, the current results demonstrate that rGO can induce cytotoxicity in MDA-MB-231 and ZR-75-1 cell lines but not in human skin fibroblasts. Furthermore, the ROS inhibitor antioxidant (NAC) decreases the cytotoxic effect of rGO in MDA-MB-231 and ZR-75-1 cells. The antiproliferative effect of rGO was associated with a decrease in MMP, increased autophagy and cell cycle arrest in breast cancer cells. The upregulation of proapoptotic protein BAX; the downregulation of antiapoptotic proteins BCL-2, BCL-xL and P65; and the subsequent activation of caspase-9, caspase-3 and cleaved-PARP in the MDA-MB-231 and ZR-75-1 cell lines, respectively, clearly indicated that the rGO induced apoptosis in the investigated cell lines. The results also provided confirmation for a role of intrinsic mechanisms in the induced apoptosis in MDA-MB-231 and ZR-75-1 cells. Furthermore, we noticed the coexistence of autophagy and apoptosis in MDA-MB-231 and ZR-75-1 cells exposed to rGO. Based on the above results, it can be concluded that apoptosis and autophagy are possible anti-cancer mechanisms underlying the cytotoxicity induced by rGO in breast cancer cells.

## Data Availability

Not applicable.

## Abbreviations

AKT	serine/threonine kinase
AMPK	5′ AMP-activated protein kinase
APAF-1	apoptotic protease activating factor 1
ATG	autophagy-related protein
BAX	Bcl-2-associated X protein
BCL-2	B-cell CLL/lymphoma 2
BCL-xL	B-cell lymphoma-extra large
BIM	Bcl-2-like protein
JC-1	5,5,6,6-tetrachloro-1,1,3,3-tetraethylbenzimidazolcarbocyanine iodide
LC3	microtubule-associated protein 1A/1B-light chain
MMP	mitochondrial membrane potential
MPTP	mitochondrial permeability transition pores
MTT	3-(4,5-dimethylthiazol-2-yl)-2,5-diphenyltetrazolium bromide
NAC	N-acetylcysteine
NF-κB	nuclear factor kappa-light-chain-enhancer of activated B cells
PARP	poly(ADP-ribose) polymerase-1
PI3K	phosphoinositide 3-kinases
rGO	reduced graphene oxide
ROS	reactive oxygen species

## References

[1] Hiroki N., Young H.K. (2017). Cancer prevention from the perspective of global cancer burden patterns. J. Thorac. Dis..

[2] Thun M.J., DeLancey J.O., Center M.M., Jemal A., Ward E.M. (2010). The global burden of cancer: Priorities for prevention. Carcinogenesis.

[3] Testa U., Castelli G., Pelosi E. (2020). Breast Cancer: A Molecularly Heterogenous Disease Needing Subtype-Specific Treatments. Med. Sci..

[4] Tuasha N., Petros B. (2020). Heterogeneity of Tumors in Breast Cancer: Implications and Prospects for Prognosis and Therapeutics. Scientifica.

[5] Jemal A., Bray F., Center M.M., Ferlay J., Ward E., Forman D. (2011). Global cancer statistics. CA Cancer J. Clin..

[6] Ou L., Lin S., Song B., Liu J., Lai R., Shao L. (2017). The mechanisms of graphene-based materials-induced programmed cell death: A review of apoptosis, autophagy, and programmed necrosis. Int. J. Nanomed..

[7] Yang K., Feng L., Liu Z. (2016). Stimuli responsive drug delivery systems based on nano-graphene for cancer therapy. Adv. Drug Deliv. Rev..

[8] Ristic B., Harhaji-Trajkovic L., Dakic M.B.I., Mijatovic S., Trajkovic V. (2021). Modulation of Cancer Cell Autophagic Responses by Graphene-Based Nanomaterials: Molecular Mechanisms and Therapeutic Implications. Cancers.

[9] Hu D., Zhang J., Gao G., Sheng Z., Cui H., Cai L. (2016). Indocyanine green-loaded polydopamine-reduced graphene oxide nanocomposites with amplifying photoacoustic and photothermal effects for cancer theranostics. Theranostics.

[10] Battogtokh G., Ko Y.T. (2016). Graphene oxide-incorporated pH-responsive folate-albumin-photosensitizer nanocomplex as image-guided dual therapeutics. J. Control. Release.

[11] Teixeira S.R., Lloyd C., Yao S. (2016). Polyaniline-graphene based α-amylase biosensor with a linear dynamic range in excess of 6 orders of magnitude. Biosens. Bioelectron..

[12] Shirai A., Henares T.G., Sueyoshi K., Endo T., Hisamoto H. (2016). Fast and single-step immunoassay based on fluorescence quenching within a square glass capillary immobilizing graphene oxide-antibody conjugate and fluorescently labelled antibody. Analyst.

[13] Lin J., Chen X., Huang P. (2016). Graphene-based nanomaterials for bioimaging. Adv. Drug Deliv. Rev..

[14] Weng X., Neethirajan S.A. (2016). Microfluidic biosensor using graphene oxide and aptamer-functionalized quantum dots for peanut allergen detection. Biosens. Bioelectron..

[15] Ali Tahir A., Ullah H., Sudhagar P., Asri Mat Teridi M., Devadoss A., Sundaram S. (2016). The application of graphene and its derivatives to energy conversion, storage, and environmental and biosensing devices. Chem. Rec..

[16] McCallion C., Burthem J., Rees-Unwin K., Golovanov A., Pluen A. (2016). Graphene in therapeutics delivery: Problems, solutions and future opportunities. Eur. J. Pharm. Biopharm..

[17] Liu H., Li T., Liu Y., Qin G., Wang X., Chen T. (2016). Glucose-reduced graphene oxide with excellent biocompatibility and photothermal efficiency as well as drug loading. Nanoscale Res. Lett..

[18] Szczepaniak J., Strojny B., Chwalibog E.S., Jaworski S., Jagiello J., Winkowska M., Szmidt M., Wierzbicki M., Sosnowska M., Balaban J. (2018). Effects of Reduced Graphene Oxides on Apoptosis and Cell Cycle of Glioblastoma Multiforme. Int. J. Mol. Sci..

[19] Hua S., Yang L., Gao T., Jiang P., Jiang F., Liu Y. (2019). Graphene Quantum Dots Induce Autophagy and Reveal Protection Against Hydrogen Peroxide-Induced Oxidative Stress Injury. ACS Appl. Bio Mater..

[20] Wang J., Wang P., He Y., Liu X., Wang S., Ma C., Tian X., Wang J., Wu X. (2020). Graphene oxide inhibits cell migration and invasion by destroying actin cytoskeleton in cervical cancer cells. Aging.

[21] Yiru Q., Zhi-Wei Z., Shu-Ting P., Zhi-Xu H., Xueji Z., Jia-Xuan Q., Wei D., Tianxin Y., Shu-Feng Z. (2015). Graphene quantum dots induce apoptosis, autophagy, and inflammatory response via p38 mitogen-activated protein kinase and nuclear factor-kB mediated signaling pathways in activated THP-1 macrophages. Toxicology.

[22] Krętowski R., Jabłońska-Trypuć A., Cechowska-Pasko M. (2021). The Preliminary Study on the Proapoptotic Effect of Reduced Graphene Oxide in Breast Cancer Cell Lines. Int. J. Mol. Sci..

[23] Minfei S., Yang M., Sangita S. (2013). Role of the Crosstalk between Autophagy and Apoptosis in Cancer. J. Oncol..

[24] Gump J.M., Thorburn A. (2011). Autophagy and apoptosis- what’s the connection?. Trends Cell Biol..

[25] Kao J., Salari K., Bocanegra M., Choi Y.L., Girard L., Gandhi J., Kwei K.A., Hernandez-Boussard T., Wang P., Gazdar A.F. (2009). Molecular Profiling of Breast Cancer Cell Lines Defines Relevant Tumor Models and Provides a Resource for Cancer Gene Discovery. PLoS ONE.

[26] Ou L., Song B., Liang H., Liu J., Feng X., Deng B., Sun T., Shao L. (2016). Toxicity of graphene-family nanoparticles: A general review of the origins and mechanisms. Part. Fibre Toxicol..

[27] Gurunathan S., Han J.W., Park J.H., Kim J.H. (2014). An in vitro evaluation of graphene oxide reduced by Gonoderma spp. In human breast cancer cells (MDA-MB-231). Int. J. Nanomed..

[28] Mendes R.G., Koch B., Bachmatiuk A., Ma X., Sanchez S., Damm C., Schmidt O.G., Gemming T., Eckert J., Rummeli M.H. (2015). A size dependent evaluation of the cytotoxicity and uptake of nanographene oxide. J. Mater. Chem. B.

[29] Wu J., Yang R., Zhang L., Fan Z., Liu S. (2015). Cytotoxicity effect of graphene oxide on human MDA-MB-231 cells. Toxicol. Mech. Methods.

[30] Alsaedi I.I.J., Taqi Z.J., Abdul Hussien A.M., Sulaiman G.M., Jabir M.S. (2019). Graphene nanoparticles induces apoptosis in MCF-7 cells through mitochondrial damage and NF-KB pathway. Mater. Res. Express.

[31] Jaworski S., Sawosz E., Kutwin K., Wierzbicki M., Hinzmann M., Grodzik M., Winnicka A., Lipińska L., Włodyga K., Chwalibog A. (2015). In vitro and in vivo effects of graphene oxide and reduced graphene oxide on glioblastoma. Int. J. Nanomed..

[32] Hinzmann M., Jaworski S., Kutwin M., Jagiełło J., Koziński R., Wierzbicki M., Grodzik M., Lipińska L., Sawosz E., Chwalibog A. (2014). Nanoparticles containing allotropes of carbon have genotoxic effects on glioblastoma multiforme cells. Int. J. Nanomed..

[33] Markovic Z.M., Harhaji-Trajkovic L.M., Todorovic-Markovic B.M., Kepić D.P., Arsikin K.M., Jovanović S.P., Pantovic A.C., Dramićanin M.D., Trajkovic V.S. (2011). In vitro comparison of the photothermal anticancer activity of graphene nanoparticles and carbon nanotubes. Biomaterials.

[34] Kang Y., Liu J., Wu J., Yin Q., Liang H., Chen A., Shao L. (2017). Graphene oxide and reduced graphene oxide induce neural pheochromocytoma-derived PC12 cell lines apoptosis and cell cycle alterations via the ERK signaling pathway. Int. J. Nanomed..

[35] Xu T., Zhang Z., Qu L. (2020). Graphene-Based Fibers: Recent Advances in Preparation and Application. Adv. Mater..

[36] Reuter S., Gupta S.C., Chaturvedi M.M., Aggarwal B.B. (2010). Oxidative stress, inflammation, and cancer: How are they linked?. Free Radic. Biol. Med..

[37] Hayes J.D., Dinkova-Kostova A.T., Tew K.D. (2020). Oxidative Stress in Cancer. Cancer Cell.

[38] Tang T., Zhao L., Yang Z., Liu Z., Gu J., Bai B., Liu J., Xu J., Yang H. (2018). Mechanisms of oxidative stress, apoptosis, and autophagy involved in graphene oxide nanomaterial anti-osteosarcoma effect. Int. J. Nanomed..

[39] Krętowski R., Borzym-Kluczyk M., Stypułkowska A., Brańska-Januszewska J., Ostrowska H., Cechowska-Pasko M. (2016). Low glucose dependent decrease of apoptosis and induction of autophagy in breast cancer MCF-7 cells. Mol. Cell. Biochem..

[40] Lammel T., Boisseaux P., Fernandez-Cruz M.L., Navas J.M. (2013). Internalization and cytotoxicity of graphene oxide and carboxyl graphene nanoplatelets in the human hepatocellular carcinoma cell line Hep G2. Part. Fibre Toxicol..

[41] Zhang B., Wei P., Zhou Z., Wei T. (2016). Interactions of graphene with mammalian cells: Molecular mechanisms and biomedical insights. Adv. Drug Deliv. Rev..

[42] Zhou H., Zhang B., Zheng J., Yu M., Zhou T., Zhao K., Jia Y., Gao X., Chen C., Wei T. (2014). The inhibition of migration and invasion of cancer cells by graphene via the impairment of mitochondrial respiration. Biomaterials.

[43] Nandi A., Ghosh C., Basu S. (2019). Polymer conjugated graphene-oxide nanoparticles impair nuclear DNA and Topoisomerase I in cancer. Nanoscale Adv..

[44] Jiang J.-H., Pi J., Jin H., Cai J.-Y. (2018). Functional graphene oxide as cancer-targeted drug delivery system to selectively induce oesophageal cancer cell apoptosis. Artif. Cells Nanomed. Biotechnol..

[45] Abbaspour Babaei M., Zaman Huri H., Kamalidehghan B., Yeap S.K., Ahmadipour F. (2017). Apoptotic induction and inhibition of NF-κB signaling pathway in human prostatic cancer PC3 cells by natural compound 2,2’-oxybis (4-allyl-1-methoxybenzene), biseugenol B, from *Litsea costalis*: An in vitro study. OncoTargets Ther..

[46] Bajpai V.K., Khan I., Shukla S., Kang S.M., Aziz F., Tripathi K.M., Saini D., Cho H.J., Heo N.S., Sonkar S.K. (2020). Multifunctional N-P-doped carbon dots for regulation of apoptosis and autophagy in B16F10 melanoma cancer cells and in vitro imaging applications. Theranostic.

[47] Kusaczuk M., Krętowski R., Bartoszewicz M., Cechowska-Pasko M. (2016). Phenylbutyrate—A pan-HDAC inhibitor—Suppresses proliferation of glioblastoma LN-229 cell line. Tumor Biol..

[48] Banerjee P.P., Bandyopadhyay A., Mondal P., Mondal M.K., Chowdhury P., Chakraborty A., Sudarshan M., Bhattacharya S., Chattopadhyay A. (2019). Cytotoxic effect of graphene oxide-functionalized gold nanoparticles in human breast cancer cell lines. Nucleus.

[49] Wang Y., Xu J. (2018). Funcjonalized graphene oxide triggers cell cycle checkpoint control through both the ATM and the ATR signaling pathway. Carbon.

[50] Hashemi E., Akhavan O., Shaamsara M., Majd S.A., Sanati M.H., Joupari M.D., Farmany A. (2020). Graphene oxide negatively regulates cell cycle in embryonic fibroblasts cells. Int. J. Nanomed..

[51] Krętowski R., Kusaczuk M., Naumowicz M., Kotyńska J., Szynaka B., Cechowska-Pasko M. (2017). The Effects of Silica Nanoparticles on Apoptosis and Autophagy of Glioblastoma Cell Lines. Nanomaterials.

[52] Bjorkoy G., Lamark T., Pankiv S., Overvatn A., Brech A., Johansen T. (2009). Monitoring Autophagic Degradation of p62/SQSTM1. Methods Enzymol..

[53] Zhang L., Ouyang S., Zhang H., Qiu M., Dai Y., Wang Y., Ou J. (2021). Graphene oxide induces dose-dependent lung injury in rats by regulating autophagy. Exp. Ther. Med..

[54] Carmichael J., DeGraff W.G., Gazdar A.F., Minna J.D., Mitchell J.B. (1987). Evaluation of a tetrazolium-based semiautomated colorimetric assay: Assessment of chemosensitivity testing. Cancer Res..

[55] Laemmli U.K. (1970). Cleavage of structural proteins during the assembly of the head of bacteriophage T4. Nature.

[56] Smith P.K., Krohn R.I., Hermanson G.T., Mallia A.K., Gartner F.H., Provenzano M.D., Fujimoto E.K., Goeke N.M., Olson B.J., Klenk D.C. (1985). Measurement of protein using bicinchoninic acid. Anal. Biochem..

